# TNFAIP9 protects against the development of the early stage of chronic kidney disease: Focus on inflammation and fibrosis

**DOI:** 10.1371/journal.pone.0325334

**Published:** 2025-06-05

**Authors:** Ying Chen, Yanqiu Li, Deyu Zhang

**Affiliations:** 1 Department of Nephrology, The First Hospital of China Medical University, Shenyang, Liaoning, China; 2 Department of Obstetrics and Gynecology, Shengjing Hospital of China Medical University, Shenyang, Liaoning, China; Jordan University of Science and Technology Faculty of Medicine, JORDAN

## Abstract

Tumor necrosis factor alpha-induced protein 9 (TNFAIP9) is a crucial effector molecule that protects cells from inflammatory and metabolic damage. This study focuses on investigating the role and regulatory mechanisms of TNFAIP9 in the progression of chronic kidney disease (CKD). By analyzing CKD-related datasets from the GEO database, we discovered that TNFAIP9 was upregulated in CKD patients and CKD mice compared to their normal controls. To elucidate the functional role of TNFAIP9, we established a mouse model of CKD through a two-step 5/6 nephrectomy (Nx). The experimental mice were transduced with an adenoviral vector to express TNFAIP9. The results showed that mice undergoing 5/6-Nx developed evident renal impairment, inflammation, and fibrosis. Overexpression of TNFAIP9 resulted in the remission of renal impairment, a decreased inflammatory response, and a reduced expression of fibrotic markers. In vitro, human renal tubular epithelial human kidney-2 (HK-2) cells were exposed to tumor necrosis factor-alpha (TNF-α) or transforming growth factor-beta (TGF-β) to simulate inflammatory and fibrotic conditions, respectively. Then, the overexpression plasmid or small interfering RNA (siRNA) targeting TNFAIP9 was transfected into HK-2 cells to either overexpress or knock down the target protein. Overexpression of TNFAIP9 reduced the TNF-α-induced inflammatory response, while its knockdown amplified it. Likewise, overexpression of TNFAIP9 decreased the TGF-β-induced fibrosis, whereas its knockdown heightened it. In summary, it is suggested that TNFAIP9 plays a protective role against the early stage of CKD by suppressing renal inflammation and fibrosis. Therefore, targeting TNFAIP9 could be a promising therapeutic approach for CKD.

## 1. Introduction

Chronic kidney disease (CKD) is defined by persistent urine abnormalities, structural abnormalities, or impaired renal excretion, indicating a loss of functional nephrons [1]. CKD typically stems from various heterogeneous diseases and ultimately progresses to end-stage renal disease (ESRD), necessitating dialysis or kidney transplantation [2]. Health services for patients with CKD are transitioning from focusing on therapeutic strategies for advanced CKD to incentivizing early detection and precise monitoring. Patients with CKD experience a reduced quality of life and worsening conditions as the disease progresses. However, the molecular mechanisms driving CKD remain elusive.

CKD is primarily characterized by injury, inflammation, and fibrosis in the kidney. Injury to the kidney initiates inflammatory and fibrotic processes to promote tissue regeneration and repair. Following renal injury, inflammatory cells multitask at the damaged site by accelerating wound debridement and releasing cytokines, chemokines, growth factors, or metabolites [3]. The normal tissue repair process typically occurs through the proper activation of fibroblasts and collagen deposition. If this well-orchestrated response is dysregulated, the activation of these inflammatory and fibrotic cells may be prolonged, leading to excessive extracellular matrix deposition and, consequently, chronic or progressive renal fibrosis [4,5]. Investigations into the mechanisms of renal inflammation and fibrosis are vital for the prevention and development of therapeutic strategies for CKD.

In recent years, the Gene Expression Omnibus (GEO) database has been widely recognized as a powerful tool for discovering disease biomarkers and potential therapeutic targets. By searching the GEO database, we found that the expression of tumor necrosis factor alpha-induced protein 9 (TNFAIP9, also known as STEAP4 or STAMP2), was significantly upregulated in CKD patients and CDK mice compared to the normal controls. TNFAIP9 is a mitochondrial metalloproteinase involved in the maintenance of iron and copper homeostasis [6]. Numerous genome-wide association studies document TNFAIP9 as a critical effector molecule involved in inflammatory and metabolic responses [7,8]. It was reported that TNFAIP9 was increased after the induction of tumor necrosis factor-α (TNF-α) and also by other inflammatory factors such as interleukin 6 (IL-6) and interleukin-1β (IL-1β) [9,10]. Other studies have reported reduced TNFAIP9 expression in response to external stimuli, such as high glucose and leptin [11]. The literature on TNFAIP9’s regulation of disease progression mostly focuses on its anti-inflammatory properties [12–14]. Several studies have highlighted the pathogenic role of TNFAIP9. For instance, TNFAIP9 downregulation was found to alleviate the lipopolysaccharide-induced inflammatory microenvironment and tumorigenesis in prostate cancer cells [15]. Additionally, TNFAIP9 knockout mice exhibited delayed onset and reduced severity of experimental autoimmune encephalomyelitis [16]. In kidney-related studies, it was proven that high glucose significantly induced TNFAIP9 expression, and overexpression of TNFAIP9 mitigated the cell damage induced by high glucose [17]. In Song et al.’s study, TNFAIP9 expression was observed to decrease in 5/6 Nx-induced CKD rats. However, omega-3 fatty acid supplementation was able to restore its expression, thereby alleviating kidney injury [18]. The expression pattern and function of TNFAIP9 in renal pathological conditions are quite controversial and require further confirmation. In this study, we conducted in vivo and in vitro experiments to investigate the expression pattern of TNFAIP9 and its potential role in the development of CKD.

## 2. Materials and methods

### 2.1 Database analysis

In order to identify differentially expressed genes (DEGs) in CKD, several datasets were analyzed in the GEO database (https://www.ncbi.nlm.nih.gov/gds). GSE66494 included 53 CKD renal biopsy specimens and 7 normal human specimens, GSE70528 had 7 CKD peripheral blood cell samples and 4 normal human samples, GSE38117 featured 3 CKD renal primary cell samples and 3 normal mouse samples, GSE148084 had 4 CKD kidney specimens and 4 normal mouse specimens, and GSE217650 contained 5 CKD kidney specimens and 5 normal mouse specimens. The online tool GEO2R was utilized individually for each dataset to pinpoint genes that showed differential expression in CKD. The standards used to identify DEGs were: log_2_FC > 1 or log_2_FC < −1, and P-value<0.01. A Venn diagram of the common DEGs was produced by the online tool JVenn (http://jvenn.toulouse.inra.fr/app/example.html). The expression pattern of the common 10 genes in five datasets was presented as a heatmap with a Z-score transformation.

### 2.2 Preparation of recombinant adenovirus

The encoding fragments of TNFAIP9 (NM_054098.3) were subcloned into an adenoviral shuttle plasmid pAdTrack-CMV (Hunan Fenghui Biotechnology Co., Ltd, China). The recombinant shuttle plasmids Ad-TNFAIP9 or its empty vector (Ad-Vector) were homologously recombined with pAdEasy-1 in *Escherichia coli* BJ5183 (Beijing Huayueyang Biotechnology Co., Ltd. China). The obtained recombinant plasmids were transfected into HEK293A cells to generate recombinant adenovirus. The titers of viruses determined: Ad-TNFAIP9 was 4.2 × 10^9 PFU/mL, while that of Ad-Vector was 4.8 × 10^9 PFU/mL.

### 2.3 Animals and surgical procedures

Animal experiments were carried out in strict accordance with the National Institutes of Health Guide for the Care and Use of Laboratory Animals and approved by the ethics committee of China Medical University (Approval No. KT2022606). Adult (8−10 weeks old) male C57BL/6J mice were obtained from Liaoning Changsheng Biotechnology Co., Ltd. The mice were acclimated for a week before undergoing a two-step 5/6 nephrectomy (Nx) procedure, which involved removing the left kidney initially and then performing contralateral pole amputation two weeks later as previously reported [19]. The surgery was performed using inhaled isoflurane anesthesia. Intraperitoneal injection of meloxicam was used for analgesia and penicillin sodium for anti-infection. Euthanasia is done by inhaling carbon dioxide. The mice were sacrificed at different time points post-5/6 Nx to examine TNFAIP9 expression patterns using western blot and immunohistochemistry, while N = 3 mice/experiment in this part. To assess the function of TNFAIP9, its expression was artificially increased using recombinant adenoviruses. Mice received injections of saline or the adenovirus (5 × 10^8^ PFU) every four weeks. Eight weeks after the surgery, urine, blood, and kidney samples were harvested for analysis. N = 6 mice/experiment in this part.

### 2.4 Measurement of 24-hour urine protein

Mice were placed in metabolic cages for 24-h urine collection. The amount of protein in a 24-h urine sample was measured using a urine protein test kit following the guidelines provided by the manufacturer (Nanjing Jiancheng Bioengineering Institute, China).

### 2.5 Measurement of blood pressure

Following urine collection, the systolic blood pressure of each mouse was measured using the ALC-NIBP noninvasive tail-cuff system (Shanghai Alcott Biotechnology Co., Ltd. China).

### 2.6 Measurement of serum creatinine and urea nitrogen

The levels of serum creatinine and urea nitrogen were measured using a creatinine assay kit (sarcosine oxidase) and a urea assay kit, respectively, following the manufacturer’s protocols from Nanjing Jiancheng Bioengineering Institute.

### 2.7 Histopathological assessment and immunostaining

Kidney tissues were fixed with 10% buffered formalin, then embedded in paraffin. Thin sections (5 micrometers thick) were stained with Hematoxylin and Eosin to observe renal damage, and with Masson’s trichrome to assess renal fibrosis. Additionally, immunohistochemistry was performed to detect the expression of TNFAIP9, neutrophil gelatinase-associated lipocalin (NGAL) and collagen III. Immunofluorescence staining was used to identify CD68. The specific antibodies used were TNFAIP9 (A17767, ABclonal Biotech Co., Ltd. China), NGAL (Ab214671, Abcam, USA), collagen III (AF5457, Affinity Biosciences, China), and CD68 (DF7518, Affinity Biosciences).

### 2.8 Western blot analysis

Protein was extracted, and Western blot analysis was performed as previously described [20]. Primary antibodies used were against TNFAIP9 (Cat. No. A17767, ABclonal Biotech Co., Ltd.), p-p65^Ser536^ (Cat. No. AF2006, Affinity Biosciences), inducible nitric oxide synthase (iNOS, Cat. No. AF0199, Affinity Biosciences), cyclooxygenase-2 (COX-2, Cat. No. AF7003, Affinity Biosciences), fibronectin (Cat. No. A12932, ABclonal Biotech Co., Ltd.), α-smooth muscle actin (α-SMA, Cat. No. AF1032, Affinity Biosciences), Collagen-III (Cat. No. AF5457, Affinity Biosciences), and β-actin (Cat. No. sc-47778, Santa Cruz, USA). The blots were developed using luminol chemiluminescence. Signal intensities from each band were quantified using ImageJ and then normalized to β-actin.

### 2.9 Quantitative real-time PCR

RNA was extracted, and quantitative real-time PCR was performed as previously described [21]. The amount of PCR product was normalized using a housekeeping gene (β-actin) to determine the relative mRNA expression of TNFAIP9. The oligonucleotide primers specific for mouse TNFAIP9 were: CCCACTGGACCAAGGAT (forward) and AGAAGACGCACAGCACA (reverse), and for human TNFAIP9 were: GATGGAGATTGGGAAAC (forward) and AGATGGCAAAGAAGTGA (reverse).

### 2.10 Measurement of TNF-α, IL-6, and monocyte chemoattractant protein-1 (MCP-1)

Levels of inflammatory cytokines TNF-α, IL-6, as well as the chemokine MCP-1, in the kidney or cell culture were measured using the respective enzyme-linked immunosorbent assay (ELISA) kits. The measurements were conducted following the protocols provided by the manufacturer (MultiSciences(Lianke)Biotech Co., Ltd.).

### 2.11 Isolation of primary mouse peritoneal macrophages

Primary mouse peritoneal macrophages were isolated as previously described [22]. The isolated mouse peritoneal macrophages were cultured in RPMI-1640 medium. The expression of TNFAIP9 was assessed through immunofluorescence staining and Western blot analysis, while the levels of TNF-α and IL-6 in cell culture were determined using ELISA.

### 2.12 Cell transfection and treatment

Human renal tubular epithelial human kidney-2 (HK-2) cells were purchased from Procell Life Science&Technology Co., Ltd (Cat. No. CL-0109). The encoding fragments of TNFAIP9 (NM_024636.3) were ligated into the pcDNA3.1 vector (Nanjing Jinsirui Biotechnology Co., Ltd.). The small interfering RNA (siRNA) targeting TNFAIP9 was designed and synthesized by JTS Scientific (Wuhan, China). Cell transfection was performed in a 6-well cell culture plate using Lipofectamine 3000. The amount of plasmid vector was 2.5 μg, while the amount of siRNA was 75 pmol. The transfection efficiency was detected by real-time PCR and western blot.

Subsequent treatment was performed 24 hours post-transfection. Cells were treated with either 20 ng/mL of TNF-α for 24 h or 5 ng/mL of transforming growth factor-β (TGF-β) for 48 h to simulate inflammation and fibrosis, respectively.

### 2.13 Statistical analysis

GraphPad Prism was used for data analysis and graph preparation. The data were expressed as mean ± SD. The statistical differences were assessed using a one-way ANOVA. One single asterisk represents a P value between 0.01 ~ 0.05. Two asterisks represent a P-value less than 0.01.

## 3. Results

### 3.1 Microarray analysis revealed that TNFAIP9 was upregulated in CKD

We obtained five datasets related to CKD from the GEO database and identified DEGs in each dataset (S1A Fig). The DEGs that were common across all five datasets were combined using a Venn diagram (S1B Fig). A total of ten genes were identified as shared among all datasets, which included *TNFAIP9, CLEC7A, TLR1, TIMP2, MARCKS, ANXA1, ST8SIA4, DOCK11, FYB,* and *CAMK1D*. A heatmap displaying the expression patterns of these genes in each dataset was generated (S1C Fig). Through a literature review on the functions of these genes, TNFAIP9 was identified as a hub gene.

### 3.2 Validation of the overexpression of TNFAIP9 in CKD mice induced by 5/6 Nx

**Fig 1:**
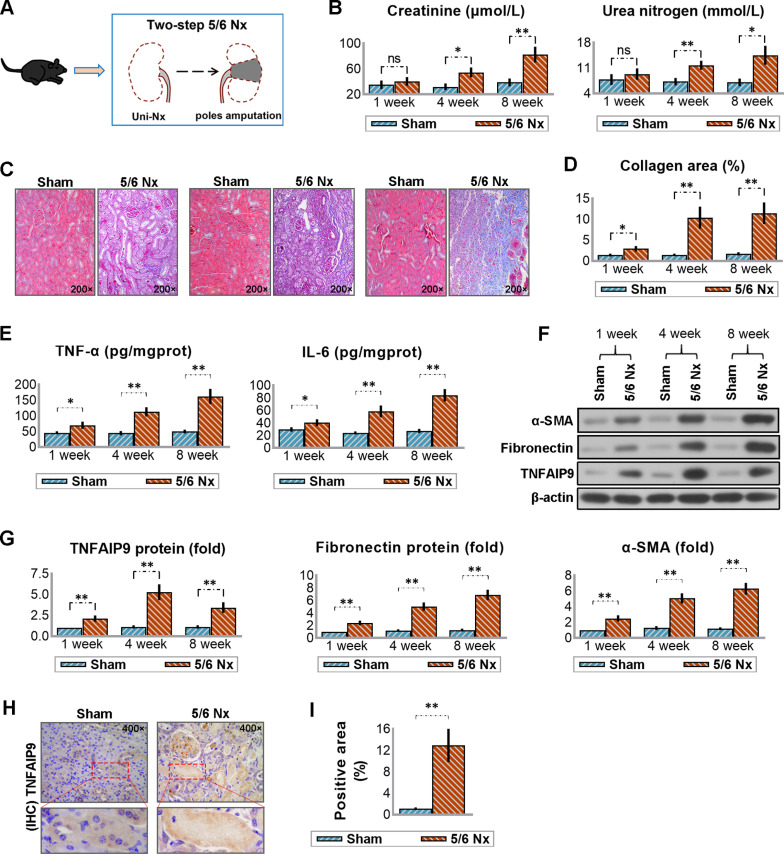
TNFAIP9 expression was increased in mice with chronic kidney disease (CKD) induced by 5/6 nephrectomy (Nx). **(A)** The study involved performing a two-step 5/6 Nx on C57 mice, which included removing one kidney and amputating the poles of the remaining kidney. **(B)** Serum creatinine and urea nitrogen levels were measured at 1, 4 and 8 weeks post-surgery. **(C)** Collagen deposition in the kidneys was assessed using Masson’s trichrome staining (×200 magnification), and **(D)** quantification of collagen content as a marker of renal fibrosis. **(E)** Levels of TNF-α and IL-6 in the kidneys were measured by ELISA. **(F)** Fibronectin, α-SMA and TNFAIP9 expression in the kidneys was analyzed by western blot followed by **(G)** quantification of the protein relative to β-actin. **(H)** Immunohistochemistry analysis of TNFAIP9 protein expression in the sections of fixed kidneys (×400 magnification) and **(I)** quantification of the positive staining area. Data were expressed as mean ± SD, with N = 3. Statistical significance was indicated by one or two asterisks denoting P values between 0.01 ~ 0.05 and less than 0.01, respectively.

To validate the TNFAIP9 expression, we constructed a mouse model with 5/6 Nx, a commonly used CKD model in rodents that closely resembles human CKD (Fig 1A). This model exhibits characteristics such as hypertension, albuminuria and glomerulosclerosis. In our study, we observed a significant increase in the levels of two kidney-excreted metabolites, creatinine and urea nitrogen, in the serum of mice following 5/6 Nx compared to Sham-operated mice. This increase was evident from the fourth week post-surgery and became more pronounced after eight weeks, indicating the persistent renal damage induced by 5/6 Nx (Fig 1B). Masson’s trichrome staining revealed collagen deposition in the kidneys of mice at 4 weeks post-5/6 Nx, which intensified by the eighth week, suggesting the development and progression of fibrosis (Fig 1C and 1D). Additionally, we examined some inflammation and fibrosis indexes, including TNF-α, IL-6, Fibronectin, and α-SMA. It was observed that the expression of these indicators gradually increased over time (Fig 1E–1G). Interestingly, the protein expression of TNFAIP9 in the remaining kidney tissue showed an early increase starting from the first week after 5/6 Nx, peaking at 4 weeks, and slightly decreasing by 8 weeks, although remaining elevated compared to Sham-operated mice (Fig 1F and 1G). Immunohistochemistry staining at the 8-week time point further confirmed this elevation (Fig 1H and 1I), indicating a potential association between TNFAIP9 expression and CKD progression.

### 3.3 Overexpression of TNFAIP9 counteracted the 5/6 Nx-induced renal injury in mice

**Fig 2:**
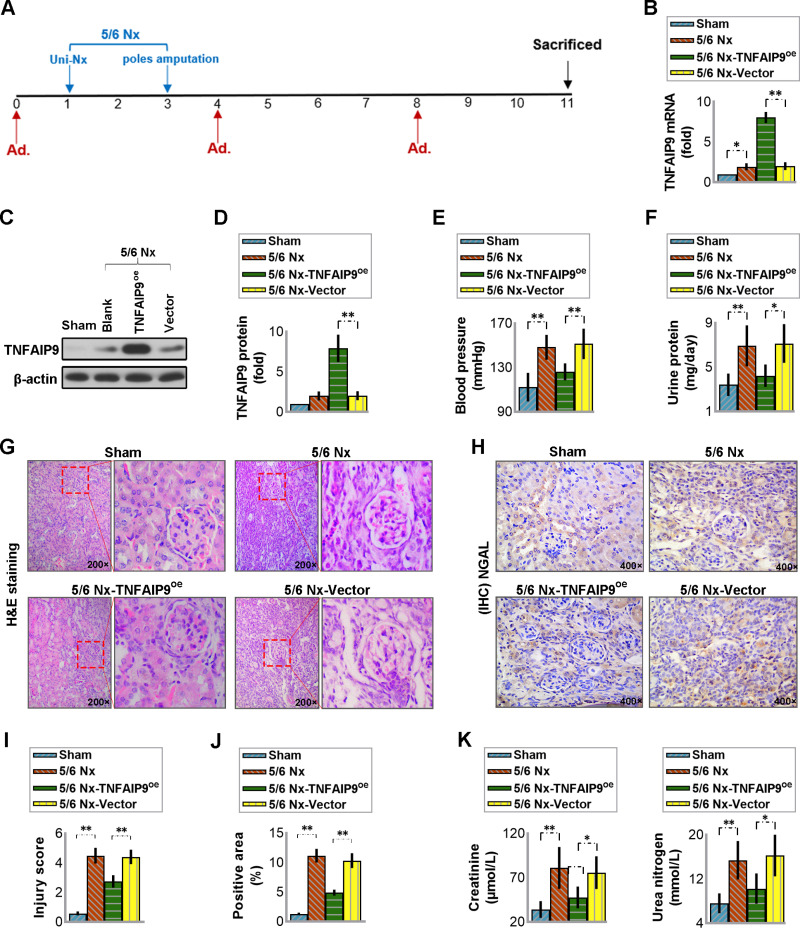
Overexpression of TNFAIP9 counteracted the renal damage caused by 5/6 Nx in C57 mice. **(A)** The mice were divided into groups and received either saline or adenovirus (Ad., 5 × 10^8^ PFU) injections every four weeks. The 5/6 Nx procedure was carried out one week after the initial adenovirus injection. After eight weeks, the 24-h urine was collected, and mice were euthanized for further analysis of serum and kidney tissues. **(B)** The mRNA expression of TNFAIP9 in the kidneys was determined by real-time PCR. **(C)** TNFAIP9 protein expression was analyzed by western blot followed by **(D)** quantification relative to β-actin. **(E)** Measurement of blood pressure in mice. **(F)** Measurement of 24-h urine protein content. **(G)** Hematoxylin and eosin staining of the kidney sections (×200 magnification) and **(I)** quantification of the kidney injury score. **(H)** Immunohistochemistry analysis of NGAL protein expression in kidney sections (×400 magnification) and **(J)** quantification of the positive staining area. **(K)** Measurement of serum creatinine and urea nitrogen levels. Data were expressed as mean ± SD, with N = 6. Statistical significance was indicated by one or two asterisks denoting P values between 0.01 ~ 0.05 and less than 0.01, respectively.

To investigate the function of TNFAIP9, we artificially overexpressed TNFAIP9 in mice with CKD model using an adenoviral vector system. The experimental procedure is outlined in Fig 2A. Confirmation of successful TNFAIP9 overexpression in the kidney was done through real-time PCR and western blot assays (Fig 2B–2D). Subsequently, we assessed the characteristic changes in CKD. Body weight in the Sham group was significantly greater than that in the other groups, and body weight in the 5/6 Nx-TNFAIP9^oe^ group tended to be greater compared with that of the Nx or Nx-Vector group (S2 Fig). Besides, mice in the 5/6 Nx group exhibited hypertension compared to the Sham group, whereas overexpression of TNFAIP9 reduced the systolic blood pressure in the 5/6 Nx-induced CKD mice compared to the vector control (Fig 2E). Also, mice in the 5/6 Nx group exhibited albuminuria and injured kidney function, as indicated by elevated serum creatinine (Fig 2K), urea nitrogen (Fig 2K), and urine protein (Fig 2F) levels. However, the upregulation of TNFAIP9 mitigated the levels of these factors (Fig 2F and 2K). Examination of kidney sections stained with Hematoxylin and Eosin revealed no apparent damage in the kidneys of Sham-operated mice. Conversely, widening of Bowman’s space in the glomeruli and significant tubular dilatation and shrinkage were observed in both the 5/6 Nx and 5/6 Nx-Vector groups. However, these pathological changes were mitigated in the 5/6 Nx-TNFAIP9^oe^ group (Fig 2G and 2I). Furthermore, immunohistochemistry staining with NGAL, a marker of renal tubular injury, indicated that staining intensity in the 5/6 Nx and 5/6 Nx-Vector groups was notably stronger than in the Sham and 5/6 Nx-TNFAIP9^oe^ groups (Fig 2H and 2J). These results suggest that TNFAIP9 overexpression protected mice from 5/6 Nx-induced renal injury.

### 3.4 Overexpression of TNFAIP9 remitted the 5/6 Nx-induced renal inflammation in mice

**Fig 3:**
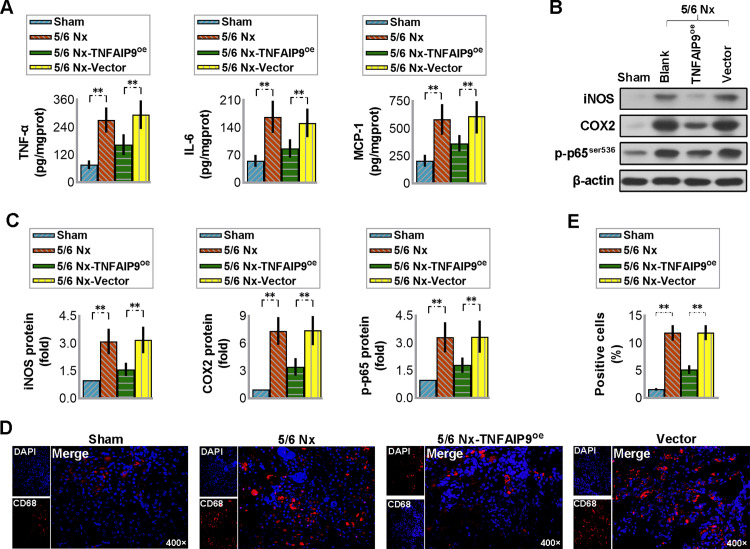
Overexpression of TNFAIP9 remitted the renal inflammation caused by 5/6 Nx in C57 mice. **(A)** Levels of TNF-α, IL-6, and MCP-1 in the kidneys were measured by ELISA. **(B)** iNOS, COX2 and p-p65^ser536^ protein expression was analyzed by western blot followed by **(C)** quantification relative to β-actin. **(D)** Immunofluorescence staining of the kidney sections for a macrophage marker, CD68 (×400 magnification) and **(E)** quantification of the positive cells. Data were expressed as mean ± SD, with N = 6. Statistical significance was indicated by one or two asterisks denoting P values between 0.01 ~ 0.05 and less than 0.01, respectively.

Inflammation serves as the early response to cellular injury, and normally leads to recovery from injury. However, persistent and uncontrolled inflammation may lead to further tissue damage. In this study, we showed that mice undergoing 5/6 Nx experienced an exacerbation of renal inflammation, as evidenced by elevated levels of inflammation-related substances such as TNF-α, IL-6, MCP-1, iNOS, COX-2, p-p65^ser636^, and CD68. Conversely, upregulation of TNFAIP9 reversed these changes (see Fig 3), suggesting that TNFAIP9 plays a role in reducing renal inflammation in mice with CKD.

### 3.5 Overexpression of TNFAIP9 alleviated the 5/6 Nx-induced renal fibrosis in mice

**Fig 4:**
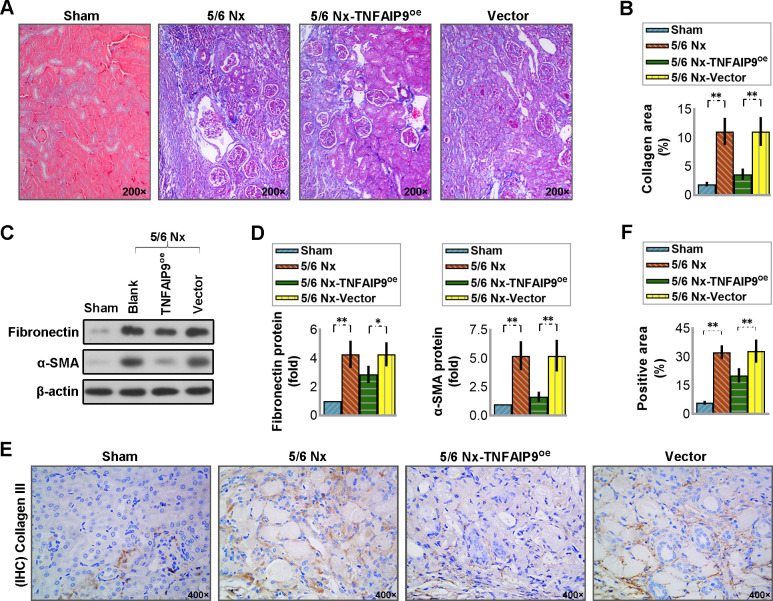
Overexpression of TNFAIP9 alleviated the renal fibrosis caused by 5/6 Nx in C57 mice. **(A)** Collagen deposition in the kidneys was assessed using Masson’s trichrome staining (×200 magnification), and **(B)** quantification of collagen content as a marker of renal fibrosis. **(C)** Fibronectin and α-SMA protein expression was analyzed by western blot followed by **(D)** quantification relative to β-actin. **(E)** Immunohistochemistry analysis of collagen III protein expression in kidney sections (×400 magnification) and **(F)** quantification of the positive staining area. Data were expressed as mean ± SD, with N = 6. Statistical significance was indicated by one or two asterisks denoting P values between 0.01 ~ 0.05 and less than 0.01, respectively.

Tubulointerstitial fibrosis is the progressive outcome and the major pathological mechanism in CKD. In this work, we observed obvious pathological deposition of collagen (a hallmark of kidney fibrosis) in mice with 5/6 Nx. Additionally, markers associated with fibrosis such as fibronectin, α-SMA and collagen III were notably elevated in the 5/6 Nx group. Conversely, mice with enhanced TNFAIP9 expression showed contrasting results (see Fig 4), indicating that TNFAIP9 augmentation may offer protection against renal fibrosis in CKD mice.

### 3.6 Overexpression of TNFAIP9 suppressed the 5/6 Nx-induced inflammation in mouse peritoneal macrophages

**Fig 5:**
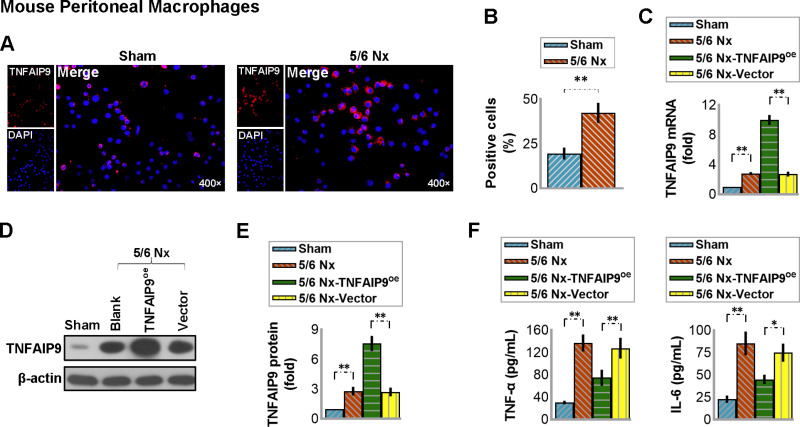
Overexpression of TNFAIP9 suppressed inflammation in mouse peritoneal macrophages caused by 5/6 Nx in C57 mice. Peritoneal macrophages were isolated from mice. **(A)** Immunofluorescence staining of the isolated macrophages for TNFAIP9 (×400 magnification) and **(B)** quantification of the positive cells. **(C)** The mRNA expression of TNFAIP9 in the isolated macrophages was determined by real-time PCR. **(D)** TNFAIP9 protein expression was analyzed by western blot followed by **(E)** quantification relative to β-actin. **(F)** Levels of TNF-α and IL-6 in the kidneys were measured by ELISA. Data were expressed as mean ± SD, with N = 6. Statistical significance was indicated by one or two asterisks denoting P values between 0.01 ~ 0.05 and less than 0.01, respectively.

There is increasing evidence suggesting that peritoneal macrophages are intensely involved in the regulation of renal inflammation [23]. Further, we wondered whether TNFAIP9 affects inflammation in peritoneal macrophages, so we isolated the peritoneal macrophages and analyzed the changes of inflammatory molecules. Our findings revealed that TNFAIP9 expression was stimulated in peritoneal macrophages of mice with 5/6 Nx, leading to a notable rise in TNF-α and IL-6 levels. Intriguingly, the systemic delivery of TNFAIP9-modified adenovirus also boosted TNFAIP9 in peritoneal macrophages while decreasing the levels of these cytokines (see Fig 5). These results indicate that overexpression of TNFAIP9 suppressed the 5/6 Nx-induced inflammation in mouse peritoneal macrophages.

### 3.7 Overexpression of TNFAIP9 inhibited TNF-α-induced inflammation in HK-2 cells

**Fig 6:**
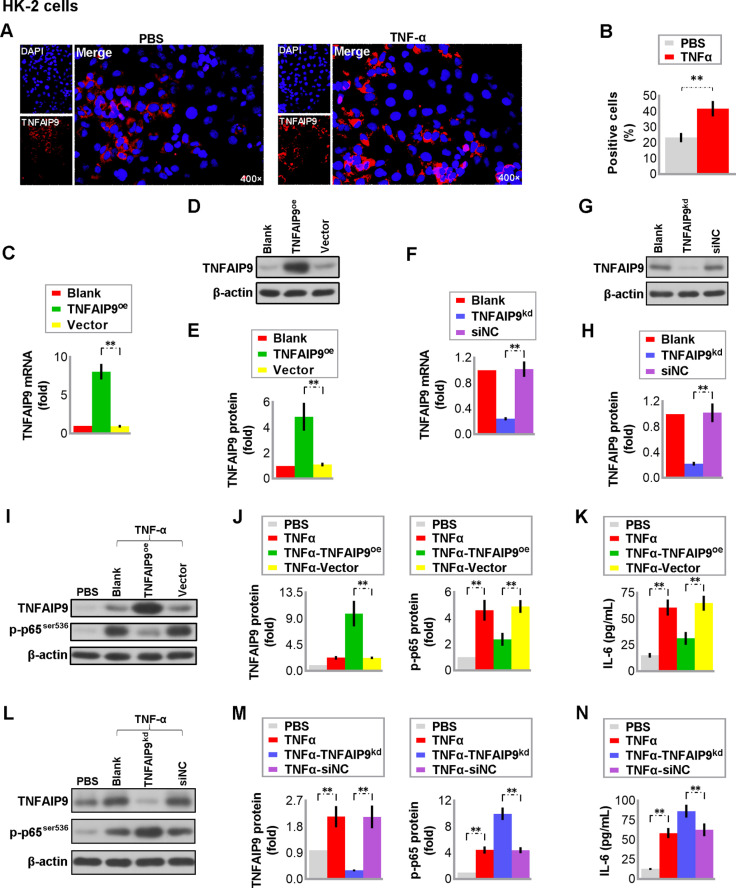
Overexpression of TNFAIP9 inhibited the TNF-α-induced inflammation in HK-2 cells. Human renal tubular epithelial HK-2 cells were treated with PBS or 20 ng/mL TNF-α for 24 h, then **(A)** immunofluorescence staining was performed for examining TNFAIP9 expression (×400 magnification) and **(B)** quantification of the positive cells. **(C-E)** HK-2 cells were transiently transfected with control vector or TNFAIP9 plasmid. Twenty-four hours later, the effect of TNFAIP9 overexpression was verified at the mRNA level by real-time PCR **(C)** and protein level by western blot **(D, E)**. **(F-H)** HK-2 cells were transiently transfected with control (NC) siRNA or TNFAIP9 siRNA. Twenty-four hours later, the effect of TNFAIP9 knockdown was verified at the mRNA level by real-time PCR **(F)** and protein level by western blot **(G, H)**. **(I-K)** HK-2 cells were transfected without or with control vector or TNFAIP9 plasmid for 24 h, then treated with either PBS or 20 ng/mL TNF-α for 24 h. **(I)** TNFAIP9 and p-p65^ser536^ expression in the cells was analyzed by western blot followed by **(J)** quantification relative to β-actin. Levels of IL-6 **(K)** in the cells were measured by ELISA. **(L-N)** Cells were transfected without or with NC siRNA or TNFAIP9 siRNA for 24 h, then treated with either PBS or 20 ng/mL TNF-α for 24 h. **(L)** TNFAIP9 and p-p65^ser536^ expression in the cells was analyzed by western blot followed by **(M)** quantification relative to β-actin. Levels of IL-6 **(N)** in the cells were measured by ELISA. Data were expressed as mean ± SD, with N = 3. Statistical significance was indicated by one or two asterisks denoting P values between 0.01 ~ 0.05 and less than 0.01, respectively.

TNF-α is an essential pro-inflammatory cytokine involved in the early inflammatory response during renal injury. Literature has shown that it could activate nuclear factor-κB (NF-κB) signaling to promote the release of pro-inflammatory factors such as IL-6 [24]. In this study, we observed that TNFAIP9 was induced in TNF-α-treated human renal tubular epithelial human kidney-2 (HK-2) cells (Fig 6A and 6B). To explore TNFAIP9’s function in vitro, HK-2 cells were transfected with a plasmid for overexpression or siRNA for knockdown. The success of overexpression or knockdown was confirmed through real-time PCR and western blot analysis (Fig 6C–6H). Overexpression of TNFAIP9 inhibited the TNF-α-induced rise in p-p65^ser635^ and IL-6 in HK-2 cells, while knockdown of TNFAIP9 increased this response (Fig 6I–6N). These results indicate that TNFAIP9 may shield HK-2 cells from the inflammatory effects of TNF-α.

### 3.8 Overexpression of TNFAIP9 inhibited the TGF-β-induced fibrosis in HK-2 cells

**Fig 7:**
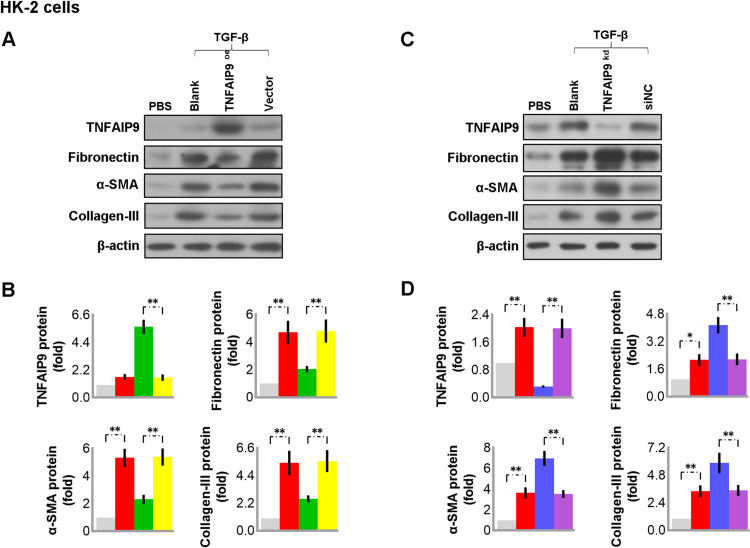
Overexpression of TNFAIP9 inhibited the TGF-β-induced fibrosis in HK-2 cells. **(A-B)** Cells were transfected without or with control vector or TNFAIP9 plasmid for 24 h, then treated with either PBS or 5 ng/mL TGF-β for 48 h. **(A)** TNFAIP9, Fibronectin, α-SMA and collagen III expression in the cells was analyzed by western blot followed by **(B)** quantification relative to β-actin. **(C-D)** Cells were transfected without or with NC siRNA or TNFAIP9 siRNA for 24 h, then treated with either PBS or 5 ng/mL TGF-β for 48 h. **(C)** TNFAIP9, Fibronectin, α-SMA and collagen III expression in the cells was analyzed by western blot followed by **(D)** quantification relative to β-actin. Data were expressed as mean ± SD, with N = 3. Statistical significance was indicated by one or two asterisks denoting P values between 0.01 ~ 0.05 and less than 0.01, respectively.

Transforming growth factor-β (TGF-β) signaling plays critical roles in renal fibrosis regulation [25]. Likewise, we found that treating HK-2 cells with TGF-β led to an increase in TNFAIP9 expression. When TNFAIP9 was overexpressed, it prevented the TGF-β-induced increase in fibronectin, α-SMA, and Collagen III levels, while reducing TNFAIP9 expression resulted in an increase in these proteins (see Fig 7). These results suggest that TNFAIP9 may have a role in inhibiting fibrosis in CKD.

## 4. Discussion

This paper discusses the expression pattern and functional role of TNFAIP9 in CKD. The results show that TNFAIP9 expression increases during the development of CKD. Overexpressing TNFAIP9 improved inflammation and fibrosis in mice with 5/6 Nx and inhibited inflammation and fibrosis in human renal tubular epithelial HK-2 cells. These findings suggest that TNFAIP9 may be induced during CKD progression due to its protective effects against renal inflammation and fibrosis.

The reduction of extracellular Fe^3+^ to Fe^2+^, and Cu^2+^ to Cu^1+^, a prerequisite for the transmembrane transport of these metals [6]. TNFAIP9 is an integral membrane metalloreductase responsible for moving electrons to extracellular iron or copper [26], and is strongly implicated in the regulation of cellular inflammation. However, the literature on the results of TNFAIP9 expression and function is quite controversial. Some studies have reported that direct exposure to pro-inflammatory cytokines increases TNFAIP9 expression [9,27,28]. In acute inflammatory conditions, there is often an elevation in TNFAIP9 levels. These elevations may be preventive [17,29,30] or promotive [7,15,16,31] to disease progression. Other reports seem counterintuitive to these studies, as TNFAIP9 expression decreases as the disease progresses, and the function of TNFAIP9 is essentially protective [12,14,18,32]. Surprisingly, the expression and function of TNFAIP9 may be altered by various pathological conditions. In this study, we found that TNFAIP9 is overexpressed in CKD using online resources. Subsequently, we experimentally demonstrated that TNFAIP9 is induced one week after 5/6 Nx in mice, and this elevation reached approximately a 5-fold increase after 4 weeks of 5/6 Nx. However, the expression of TNFAIP9 decreased slightly at 8 weeks after surgery compared to 4 weeks, but it was still at a higher level than the Sham. The reason for this phenomenon is that TNFAIP9, as an anti-inflammatory molecule, is largely produced to defend against excessive inflammatory responses in the early stages of CKD. However, the chronic and persistent inflammation of CKD may, in turn, impair TNFAIP9, resulting in a partial decrease in TNFAIP9 expression.

Renal inflammation arises as a preventive mechanism to fight against early kidney injury and to facilitate repair. However, if these reparative processes fail, the inflammatory response can become persistent and deleterious, eventually leading to malignant consequences for the tissue [33]. In CKD, there is usually a persistent activation of inflammation, characterized by the recruitment of immune cells and inflammatory cytokines, chemokines, and growth factors in the kidney. This altered microenvironment leads to an overproduction of pro-inflammatory and pro-fibrotic factors, resulting in progressive nephron loss, glomerular and interstitial fibrosis, ultimately leading to ESRD and/or premature death [34]. Macrophages, a type of innate immune cells, are predominantly present in the renal parenchyma in response to kidney injury. They could be divided into two broad groups: resident tissue macrophages and inflammatory macrophages. Tissue-resident macrophages are heterogeneous and play a role in establishing and maintaining tissue homeostasis. Inflammatory macrophages are mainly derived from the circulating environment and can infiltrate the damaged tissue to produce inflammatory factors [35]. Much of the available evidence indicates that signals originating from the microenvironment of the damaged kidney trigger the activation of macrophages [36]. In line with the previous study [37], we observed an inflammatory response in the remaining kidney of 5/6 Nx-induced CKD mice in this study. Likewise, we also observed an inflammatory response in the peritoneal macrophages. Notably, we discovered that the exogenous boost of TNFAIP9 not only attenuated the inflammatory response in the kidney but also in the circulating macrophages of CKD mice. An in vitro validation experiment also demonstrated that human renal tubular epithelial cells exhibited an inflammatory response when stimulated by the pro-inflammatory factor TNF-α, and the overexpression of TNFAIP9 reversed this phenomenon. These findings suggest that TNFAIP9 serves as a critical protective mechanism against inflammation as CKD progresses.

As is well known, sterile inflammation plays a prominent role in initiating renal fibrosis. The pro-inflammatory molecules secreted by inflammatory cells, such as MCP-1, TGF-β and platelet-derived growth factor [4], are known as critical inducers in initiating fibrosis. Tubulointerstitial fibrosis is a progressive process that affects the kidneys and is a major pathological mechanism in CKD regardless of cause [38]. The hallmark of renal fibrosis, similar to other tissues or organs, is the formation and accumulation of extracellular matrix [39]. The fibrotic process frequently results in organ deterioration and is linked to high morbidity and mortality [40]. In this study, we observed significant collagen deposition and changes in fibrosis-related factors in the kidneys of mice with CKD. Administration of the TNFAIP9-overexpressed adenovirus vector ameliorated the fibrosis in the residual kidney. An *in vitro* experiment with TGF-β stimulation in HK-2 cells showed that TNFAIP9 has the potential to alleviate fibrotic changes. This indicates TNFAIP9 as a crucial effector molecule in the defense against fibrosis in CKD.

Unfortunately, there are some limitations to the study. Firstly, the exact timing of TNFAIP9 expression changes in CKD is not known as the study only looked at expression changes at one, four, and eight weeks post-nephrectomy. It would be beneficial to examine smaller time intervals and longer time points. Secondly, TNFAIP9 expression still showed an increase at the eighth week after nephrectomy. Conducting further experiments to knock down TNFAIP9 expression in vivo could provide a clearer understanding of its role. Thirdly, while TNFAIP9 influences the direction of inflammation and fibrosis, the specific factors and pathways it targets have not been identified. Despite these limitations, the study was the first to suggest that TNFAIP9 might serve as a compensatory mechanism to up-regulate and protect kidney tissue from inflammation and fibrosis, ultimately slowing down the progression of CKD.

In conclusion, this study suggests that TNFAIP9 is up-regulated and plays a protective role against the early phase of CKD by suppressing renal inflammation and fibrosis. As such targeting TNFAIP9 may be an appealing therapeutic target for CKD.

## Supporting information

S1 Fig Microarray analysis from GEO database showed TNFAIP9 was enhanced in CKD patients and mice. **(A)** Five datasets related to CKD, including two datasets of Homo sapiens and three datasets of Mus musculus, were retrieved from the GEO database for analysis. The common DEGs in the five series were screened. The standard set for the DEGs was log|FC| > 1 and p-value < 0.01. **(B)** Left: Venn diagram illustrating the DEGs in CKD from five GEO series. Upper right: A histogram shows the number of DEGs in each dataset. Bottom right: Number of DEGs shared by 1, 2, 3, 4, and 5 datasets. The common DEGs across the five datasets were: TNFAIP9, CLEC7A, TLR1, TIMP2, MARCKS, ANXA1, ST8SIA4, DOCK11, FYB and CAMK1D. **(C)** Heatmap of the common DEGs in five datasets.
(TIF)

S2 Fig Overexpression of TNFAIP9 inhibits the weight loss caused by 5/6 Nx in C57 mice. The body weight was recorded from week 1 to week 11 of the experiment.
(TIF)
