# Supplementary figures and images for: TNFAIP9 protects against the development of the early stage of chronic kidney disease: Focus on inflammation and fibrosis
Source: PLoS One. 2025 Jun 5;20(6):e0325334. doi: 10.1371/journal.pone.0325334 (PMC12140252; doi:10.1371/journal.pone.0325334)

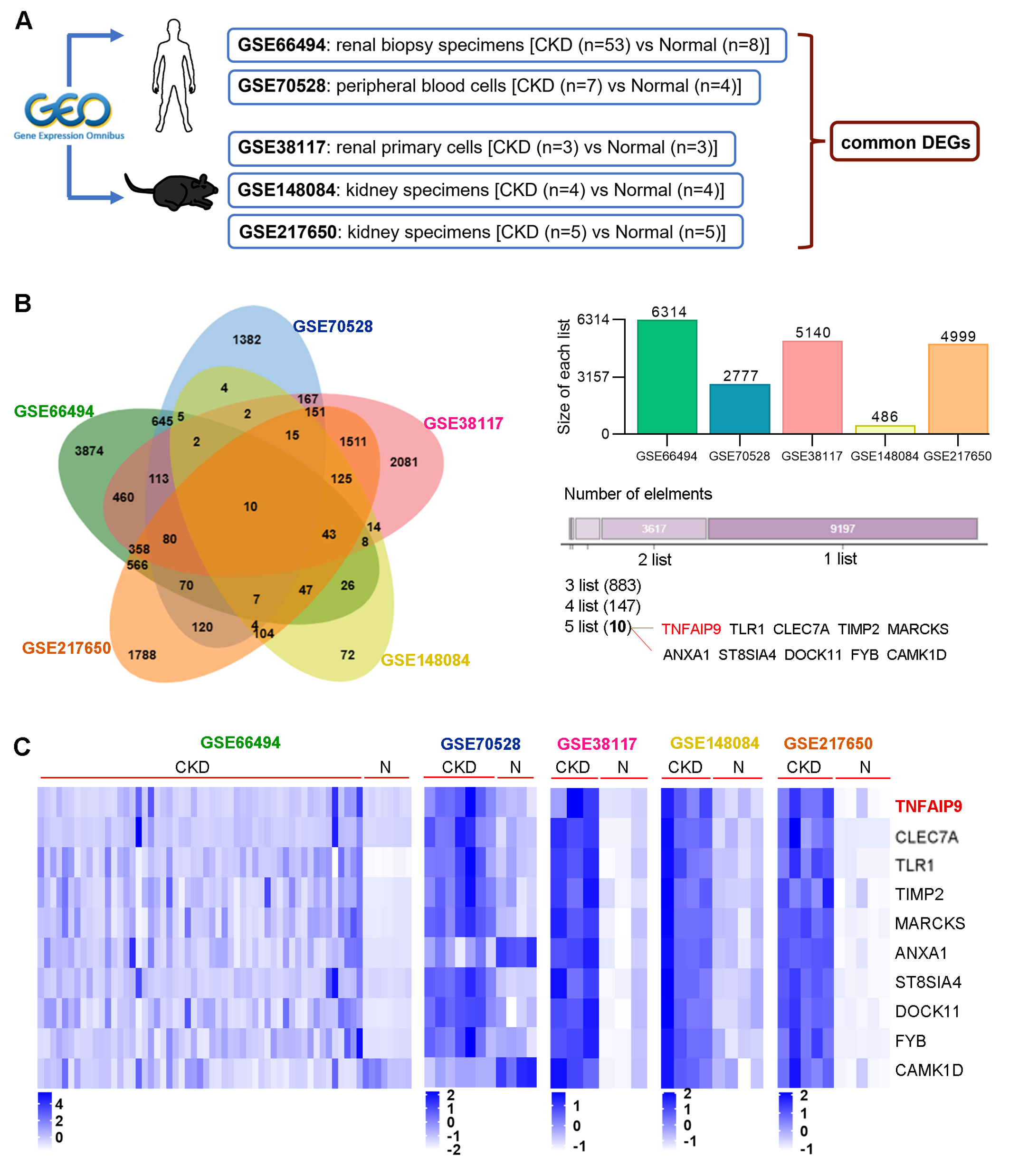

Supplement: S1 Fig — (A) Five datasets related to CKD, including two datasets of Homo sapiens and three datasets of Mus musculus, were retrieved from the GEO database for analysis. The common DEGs in the five series were screened. The standard set for the DEGs was log|FC| > 1 and p-value < 0.01. (B) Left: Venn diagram illustrating the DEGs in CKD from five GEO series. Upper right: A histogram shows the number of DEGs in each dataset. Bottom right: Number of DEGs shared by 1, 2, 3, 4, and 5 datasets. The common DEGs across the five datasets were: TNFAIP9, CLEC7A, TLR1, TIMP2, MARCKS, ANXA1, ST8SIA4, DOCK11, FYB and CAMK1D. (C) Heatmap of the common DEGs in five datasets. (TIF) [file pone.0325334.s001.tif]

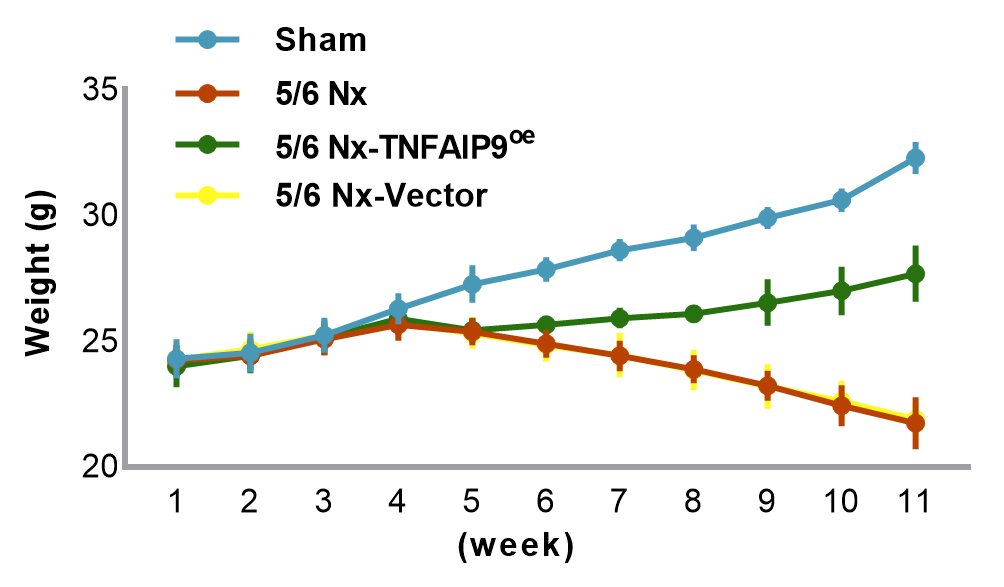

Supplement: S2 Fig — The body weight was recorded from week 1 to week 11 of the experiment. (TIF) [file pone.0325334.s002.tif]
